# Crystal Structure of the Fibre Head Domain of the Atadenovirus Snake Adenovirus 1

**DOI:** 10.1371/journal.pone.0114373

**Published:** 2014-12-08

**Authors:** Abhimanyu K. Singh, Rosa Menéndez-Conejero, Carmen San Martín, Mark J. van Raaij

**Affiliations:** 1 Departamento de Estructura de Macromoleculas, Centro Nacional de Biotecnologia (CNB-CSIC), Madrid, Spain; 2 NanoBiomedicine Initiative, Centro Nacional de Biotecnologia (CNB-CSIC), Madrid, Spain; French National Centre for Scientific Research, France

## Abstract

Adenoviruses are non-enveloped icosahedral viruses with trimeric fibre proteins protruding from their vertices. There are five known genera, from which only Mastadenoviruses have been widely studied. Apart from studying adenovirus as a biological model system and with a view to prevent or combat viral infection, there is a major interest in using adenovirus for vaccination, cancer therapy and gene therapy purposes. Adenoviruses from the Atadenovirus genus have been isolated from squamate reptile hosts, ruminants and birds and have a characteristic gene organization and capsid morphology. The carboxy-terminal virus-distal fibre head domains are likely responsible for primary receptor recognition. We determined the high-resolution crystal structure of the Snake Adenovirus 1 (SnAdV-1) fibre head using the multi-wavelength anomalous dispersion (MAD) method. Despite the absence of significant sequence homology, this Atadenovirus fibre head has the same beta-sandwich propeller topology as other adenovirus fibre heads. However, it is about half the size, mainly due to much shorter loops connecting the beta-strands. The detailed structure of the SnAdV-1 fibre head and other animal adenovirus fibre heads, together with the future identification of their natural receptors, may lead to the development of new strategies to target adenovirus vectors to cells of interest.

## Introduction

Adenoviruses can cause respiratory disease, eye disease, myocarditis and gastro-enteritic disease (among others) and have been studied as a molecular biology model system [1], for instance mRNA splicing was first observed in adenovirus-infected cells. Adenoviruses are icosahedral, non-enveloped particles [2]. Each icosahedral facet is formed by twelve hexon trimers [3], while a pentameric penton base sits at each of the vertices [4]. Trimeric fibre proteins protrude from each of the twelve vertices. These fibre proteins have an amino-terminal virus-binding sequence, a central shaft domain [5], and a globular, carboxy-terminal fibre head domain [6], [7] involved in initial receptor-binding. Depending on the adenovirus species, the fibre head may recognize one or more cell surface receptors such as the coxsackievirus and adenovirus receptor (CAR) [8], [9], [10], sialic acid [11], CD46/80/86 [12], [13] and desmoglein 2 [14]. This initial interaction is then followed by the penton base binding to cell surface integrins, leading to viral internalization via endocytosis [15].

The *Adenoviridae* family contains five genera: Mastadenovirus (all hitherto discovered human adenovirus types and most mammalian adenoviruses are Mastadenoviruses), Aviadenovirus (two-fibred adenoviruses exclusive to birds [16]) and the newly identified genera Atadenovirus, Siadenovirus and Ichtadenovirus [17]. Although structures of fibre proteins from canine Mastadenovirus 2 [18], [19] and a fowl Aviadenovirus [20], [21] have been determined, the fibres from Atadenoviruses, Siadenoviruses and Ichtadenoviruses are neither structurally nor functionally characterized.

Apart from the interest in adenovirus as disease-causing agents and for vaccination purposes, adenoviruses are also used as a framework for developing vectors to be used in gene and cancer therapy, thanks to their distinctive features such as a high potential trans-gene capacity, possibility of recombinant growth up to high titre, and efficient transduction [22], [23]. A potential downside of using vector systems based on human adenoviruses is that patients may have pre-established immunity against them. Use of distantly related non-human adenoviruses might be advantageous, as they may be less immunogenic and may have novel receptor-binding properties [24]. Recently we reported electron microscopy studies on Lizard Atadenovirus 2 [25] and crystallization of the fibre heads of Snake Atadenovirus 1 [26] and Turkey Siadenovirus 3 [27].

Snake Atadenovirus 1 (SnAdV-1) was first isolated from a corn snake (*Elaphe guttata*) with clinical signs of pneumonia [28] and classified as an Atadenovirus based on its sequence (GenBank entry DQ1064141.1; [29]). Like most structural proteins, the gene coding for the fibre protein is preserved in SnAdV-1, although we previously showed it encodes a 345 amino acid protein, rather than 415 as reported [26]. Sequence analysis suggested residues 234–345 may form a virus-distal, globular, carboxy-terminal head domain [26]. However, the putative SnAdV-1 fibre head domain has a very low sequence similarity with adenovirus fibre heads of known structure (between 12 and 18% sequence identity according to the EMBOSS STRETCHER program [30]). The Ovine Atadenovirus (OvAd-D) fibre head is also significantly smaller than other adenovirus fibre heads, as shown by electron microscopy [31].

In this work, we present the structure of the SnAdV-1 fibre head domain solved by X-ray crystallography, using the multi-wavelength anomalous dispersion (MAD) method. This is the first Atadenovirus for which the structure of the fibre head has been determined. Knowledge of the structure will lead to better understanding of the infection mechanism of the virus and may allow the design of chimeric adenoviruses with novel cell targeting properties.

## Materials and Methods

Expression of N-terminally six-histidine tagged SnAdV-1 fibre protein constructs 171–345 and 234–345 in *Escherichia coli*, purification by nickel affinity chromatography followed by strong anion exchange chromatography and crystallization by sitting drop vapour diffusion have been reported before [26]. Native crystallographic data were collected from three different crystals containing residues 234–345 (two different *P*2_1_2_1_2_1_ space group crystal forms and one *I*2_1_3 space group crystal form) and, later, from one crystal containing amino acids 171–345 (*F*23 space group crystal form). As the native protein did not contain methionine residues in the 234–345 stretch, leucines 322 and 324 were mutated to methionine for selenomethionine derivatization [26]. *De novo* structure solution by MAD (multi-wavelength anomalous dispersion) was performed using a multi-wavelength dataset obtained for selenomethionine-containing crystals of the *I*2_1_3 space group from 234–345 construct. The AUTOSHARP pipeline [32] was used for structure solution, incorporating SHELX [33] for location of heavy atom sites, SOLOMON [34] for solvent correction and ARP-WARP [35] for density interpretation and automated model building. Eight selenium sites were found, compatible with four monomers per asymmetric unit and each monomer containing two selenomethionines, as expected (for phasing statistics, see Table 1). Model completion was done with COOT [36] and the structure refined against both the native and selenomethionine data with REFMAC [37], selecting the same reflections from both datasets for calculation of Rfree [38]. The final structures had good geometric parameters and could be refined to low R-factors (Table 1). In the structures where non-crystallographic symmetry (NCS) was present, reflections for calculation of Rfree were selected in thin shells (SFTOOLS, CCP4 [39]).

**Table 1:** Crystallographic structure solution and refinement (1 Å = 0.1 nm).

					
Phasing	Remote	Peak	Inflection point	Overall	
Wavelength	0.9768	0.9791	0.9793		
Resolution rangeused (Å)	29.3–1.6	29.3–1.9	29.3–1.9		
Reflections(acentric/centric)	69500/2391	41317/2402	41283/2374		
Heavy atom sites				8 Se	
Correlationcoefficient (all/weak)				60.91/52.64	
Patterson figure ofmerit				16.56	
Correlationcoefficient (E)				0.560	
R-cullisisomorphous(acentric/centric)	0.646/0.616	0.686/0.665	−/−		
R-cullis anomalous(acentric)	0.641	0.455	0.518		
Phasing powerisomorphous(acentric/centric)	1.378/0.886	0.864/0.466	−/−		
Phasing poweranomalous(acentric)	1.899	2.665	2.356		
FOM^a^ (acentric/centric)				0.554/0.357	
**Solvent flattening** b					
R-factor(before/after)				0.3720/0.1560	
Correlation on |E|^2^(before/after)				0.5492/0.9063	
Correlation on|E|^2^/contrast(original/inverted)				0.1248/1.2247	
**Refinement**					
	Selenomethionine	Native 1	Native 2	Native 3	Native 4
Space group	*I*2_1_3	*P*2_1_2_1_2_1_	*P*2_1_2_1_2_1_	*I*2_1_3	*F*23
Cell parameters(a, b, c, Å)	149.5, 149.5,149.5	79.6, 122.3,133.7	96.8, 96.8, 153.3	149.6, 149.6,149.6	121.5, 121.5, 121.5
Monomers/asymmetric unit	4	12	12	4	1
Resolution (Å)	29.3–1.6 (1.70–1.60)^c^	29.9–2.7(2.86–2.70)	29.7–1.9 (2.03–1.90)	29.3–1.7 (1.80–1.70)	43.0–1.33 (1.37–1.33)
Reflections used	70937 (11269)	32882 (5194)	111478 (19922)	59326 (9523)	32152 (2285)
Reflections used for*R*_free_	1975 (362)	2057 (329)	2003 (278)	1604 (233)	1832 (138)
*R*-factor^d^	0.158 (0.190)	0.212(0.316)	0.173 (0.212)	0.167 (0.226)	0.117 (0.210)
*R*-free	0.189 (0.192)	0.247(0.340)	0.239 (0.250)	0.201 (0.239)	0.138 (0.225)
Number ofatoms	3927	9799	10974	4006	1019
Average B/Wilson B(Å^2^)	18.4/13.9	49.5/55.1	32.1/25.1	19.1/16.5	22.1/15.6
Ramachandran statistics (%)	98.9/100.0	96.7/99.9	98.5/100.0	98.8/100.0	100.0/100.0
r.m.s.d.bonds (Å)/angles (°)	0.012/1.5	0.016/1.3	0.014/1.4	0.013/1.5	0.013/1.6
PDB code	4D0U	4D1F	4D1G	4D0V	4UMI

a Figure of merit = cos(phase error).

b 41.5% solvent content.

c Values in parentheses are for the highest resolution bin (where applicable).

d R_symm_ = Σ_h_Σ_i_|I_hi_-<I_h_>|/Σ_h_Σ_i_|I_hi_|, where I_hi_ is the intensity of the *i*th measurement of the same reflection and <I_h_> is the mean observed intensity for that reflection.

The structures of *P*2_1_2_1_2_1_ crystal forms and *F*23 crystal form were solved by molecular replacement using PHASER [40] and refined. All refined crystallographic models were validated with MOLPROBITY [41]. The two different crystal forms belonging to the orthorhombic space group *P*2_1_2_1_2_1_ both had four trimers in the asymmetric unit. The purified 171–345 construct protein crystallized in space group *F*23 after a period of about one year and density for residues 171–231 was absent, indicating a possible proteolysis event during crystallization. Refinement statistics for all models are given in Table 1. Coordinate files and structure factors will be available from the PDB under codes 4C0U (*I*2_1_3 selenomethionine derivative), 4D1F (first *P*2_1_2_1_2_1_ crystal form), 4D1G (second *P*2_1_2_1_2_1_ crystal form), 4D0V (*I*2_1_3 native) and 4UMI (*F*23 crystal form). Structure superpositions were performed using the secondary structure element matching option of CCP4MG [42]. Structure figures were prepared with PyMOL (The PyMOL Molecular Graphics System, Version 1.5.0.4. Schrödinger, LLC).

## Results and Discussion

The structure of the SnAdV-1 fibre head domain was determined by multi-wavelength anomalous diffraction up to 1.6 Å resolution, using data collected from a selenomethionine derivative crystal as described [26]. Four monomers were present in the asymmetric unit, three (chains A, B and C) forming the biological trimer and the other (chain D) forming a trimer with two crystallographically equivalent monomers in the crystal (space group *I*2_1_3 has a three-fold crystallographic symmetry axis). Phasing statistics were very good (Table 1). Amino acids from the N-terminal T7 and 6xHis tags could not be modelled in the structure due to lack of density, as was also the case for residues 234–236 of each of the chains and residue Lys345 of chains A, B and C. Using NCS-restraints, the structures of both the seleno-methionine variant and native protein could be refined to low R-values at 1.6 and 1.7 Å resolution, respectively, with good geometry and Ramachandran statistics (Table 1). The head domain, with its eight-stranded beta-sandwich, starts at residue 238.

The structures of *P*2_1_2_1_2_1_ crystal forms were solved by molecular replacement using a trimer of the determined structure as a template and refined using NCS-restraints at 2.7 and 1.9 Å resolution, respectively. The two different crystal forms belonging to the orthorhombic space group *P*2_1_2_1_2_1_ both had four complete trimers in the asymmetric unit.

The purified 171–345 construct protein crystallized in space group *F*23 after a period of about one year. After structure solution by molecular replacement, it was noted that density for residues 171–231 was absent. The asymmetric unit of the crystals contains one monomer of the SnAdV1 fibre head, with all the residues of the fibre head (Pro232 to Lys345) being well-resolved. Coordinates and individual atom anisotropic temperature factors were refined at 1.33 Å resolution, leading to excellent agreement of the model with the data.

The fact that density for residues 171–231 was absent even when maps were calculated using low-resolution reflections only, combined with the long lag-time before crystallization, indicates a possible proteolysis event during crystallization. Two other observations support this hypothesis. The first is that there is no room in the crystal lattice at the bottom of the head domain to accommodate a shaft domain (S1 Figure); the second is that residues 232–234 point away from the three-fold trimer axis and away from each other, instead of downwards towards a potential shaft domain. The electron density for these residues is clear enough to make this interpretation reliable, while the low refinement R-factors suggests we are not missing a major part of the structure in our crystallographic model. Disorder predictions by the DISEMBL program [43] suggest that residues 230–236 have a higher probability of being disordered (S2 Figure), which also suggests the head-shaft junction of the fibre may be flexible and more accessible to proteases than the head domain.

When the structures of the fibre head monomer of the different crystal forms are superimposed, no significant differences in main chain conformations were observed. The r.m.s.d. (root mean square difference) between the structures is less than 0.38 Å and even the loop regions superimpose very well, suggesting little flexibility. When trimers are superimposed, the r.m.s.d. is less than 0.5 Å, suggesting the orientation of the monomers in the trimer is also fixed. Given the structural similarity between the crystal forms, the description below is based on the highest-resolution structures obtained, but is general to all structures.

**Figure 1:**
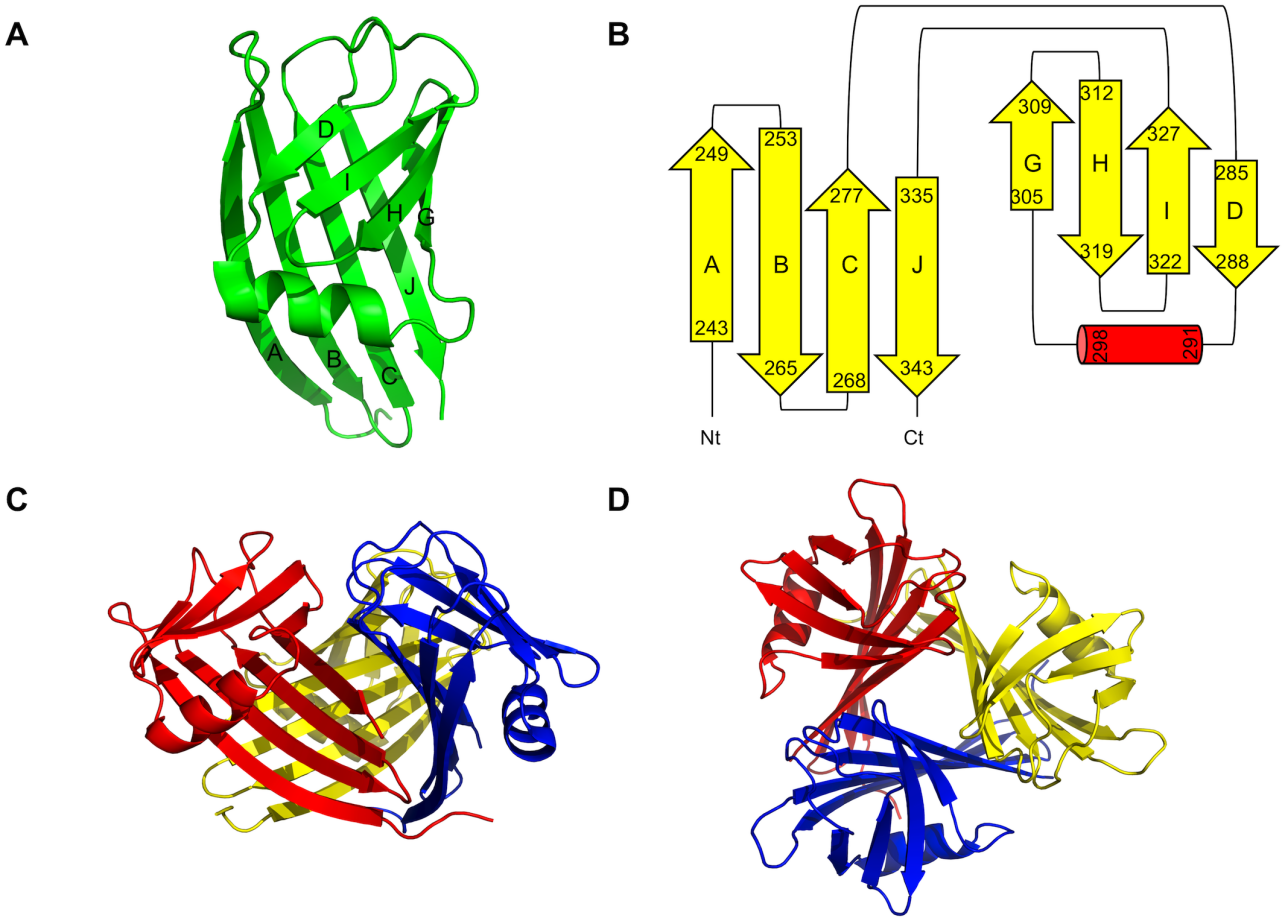
Structure of the Snake Atadenovirus 1 fibre head. A. Monomer structure. The beta-strands are labeled. B. Topology. The ABCJ and GHID beta-sheets are coloured yellow, the alpha-helix in the DG-loop is shown in red. C. Trimer structure, side view, with the three monomers coloured differently. D. Trimer structure, top view.

**Figure 2:**
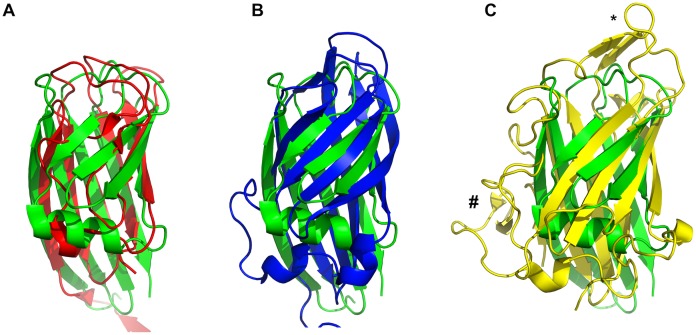
Structural comparison with other viral receptor-binding proteins. Superposition of the SnAdV-1 fibre head monomer structure (green) onto the bacteriophage TP901-1 receptor-binding protein monomer (A; PDB entry 2F0C; red), onto the avian reovirus fibre head domain monomer (B; PDB entry 2BT7; blue) and the HAdV-37 fibre head monomer (C; PDB entry 2WBW; yellow). The SnAdV-1 fibre head monomer is in the same orientation as in Fig. 1A. The top of the trimer is indicated with * and the side with #.

The SnAdV-1 fibre head is the first Atadenovirus protein structure to be solved at atomic resolution (Fig. 1). The head domain starts at residue 238 and each monomer contains an eight-stranded beta-sandwich. Despite the low sequence identity with other fibre heads, the topology of the beta-sandwich is identical to that of known adenovirus fibre head structures and contains ABCJ and GHID beta-sheets (the E- and F-strands in the long DG-loop of human adenoviruses 5 and 2 [6], [7] are absent). However, it is significantly more compact (Fig. 2C), making the 107-amino acid long SnAdV-1 fibre head the smallest adenoviral fibre head structure known. It has a diameter of 4.6 nm and a height of 3.8 nm (compared to a diameter of 6.2 nm and a height of 4.0 nm for the HAdV-5 fibre head [6]). The SnAdV-1 fibre head may be considered a “minimal” adenovirus fibre head fold, although smaller fibre trimerization domains are known, such as the bacteriophage T4 foldon [44]–[45].

In the trimer, the ABCJ-sheet is partially buried, facing the inside, while the GHID-sheet is mainly solvent-exposed. This means that the ABCJ-sheet contributes the majority of inter-monomer contacts. The strands making up the buried sheet are longer than those of the exposed sheet (average of ten residues per strand *vs*. six). The beta-strands in the structure are connected by either loops or beta-turns. Seven such connections exist; three loops (CD, DG and IJ) of variable size and four beta-turns (AB, BC, GH and HI) of two residues each. The CD and IJ loops contain 7 residues each, while the DG loop is longer, 16 residues. In SnAdV-1, an alpha-helix of eight residues is present in the DG-loop, rather than beta-strands like in Human Adenovirus 5.

As mentioned, the fibre head of SnAdV-1 has little sequence identity with fibre heads of known structure. However, fibres from other adenoviruses, from reoviruses as well as receptor-binding proteins from bacteriophages were identified with a similar fold (Fig. 2). A DALI search [46] showed receptor binding protein of bacteriophages TP901-1 [47] and p2 [48] as the closest match with Z-values of around 7 (Fig. 2A). According to Holm et al. [46], significant similarities have a Dali Z-score above 2 and they usually correspond to similar folds. Strong matches have a Z-score above n/10−4, where n is the number of residues in the query structure, giving a Z-value cut-off of around 6 in this particular case. Further hits included the mammalian and avian reovirus attachment proteins sigma1 [49] and sigmaC [50], [51]. All these proteins share the same ABCJ-GHID beta-sandwich topology. An evolutionary relationship between adenovirus and reovirus fibres has been proposed before, based on structural similarity between their triple beta-spiral repeat shaft and beta-structured head domains [5], [49]. Strengthening this idea, the SnAdV-1 fibre head has close similarity, more than any other adenovirus heads, to reovirus attachment proteins sigma1 and sigmaC. However, sigma1 and sigmaC have a circular arrangement of the beta-structured head domain, a “beta-barrel”, with strands C and H carrying kinks, whereas SnAdV-1 fibre head has a sandwich like appearance with no observable kinks in any of its strands (Fig. 2B).

Spinelli *et al*. proposed an evolutionary relationship between phage receptor-binding domains and adenovirus and reovirus fibre head domains [48]. The small (just under 100 residues per monomer), trimeric, C-terminal receptor binding domains of TP901-1 and p2 bacteriophages are beta-barrels made up of six and seven anti-parallel beta-strands, respectively. Their compact structures are comparable to the SnAdV-1 fibre head in dimensions and secondary structure, despite having fewer beta-strands (Fig. 2A).

Among adenovirus fibre head domains, SnAdV-1 fibre head is most similar to the fibre heads of Porcine Adenovirus 4 [52] and Human Adenoviruses 19p and 37 [53]. All of these structures have a similar beta-sandwich arrangement, containing ABCJ- and GHID-sheets, but unlike the SnAdV-1 fibre head, structures HAdV-19p and HAdV-37 have longer loops connecting the beta-strands. Human Adenovirus 19p and 37 fibre head bind both the Coxsackievirus and Adenovirus Receptor protein (CAR) and/or sialic acid [19]. However, in the SnAdV-1 fibre head domain, the loops that interact with CAR (on the side of the trimer) or sialic acid (on the top of the trimer) are significantly shorter and have different conformations (Fig. 2C). Also, unlike fibre heads of HAdV-37 and HAdV-19p, which have a high predicted pI, the predicted pI of SnAdV-1 fibre head is around 5.

The trimeric packing of SnAdV-1 fibre head is highly stable, exemplified by the fact that to visualize monomers on a sodium dodecylsulphate-gel, boiling the protein in protein sample buffer containing dodecylsulphate for a few minutes is necessary before sample loading [26]. This is also true for other virus and phage fibre proteins [54]. Each monomer has a total solvent accessible surface area of 5.9×10^3^ Å^2^, out of which 1.4×10^3^ Å^2^ (24%) gets buried upon trimer formation. Eleven hydrogen bonds and ten salt bridges are contributed by each monomer to the assembly. The majority of these residues responsible for inter-monomer interactions are located on strands B, C and J. Approximately 8.5 kcal/mol of energy is required to dissociate the trimer into monomers, as calculated by PISA [55].

**Figure 3:**
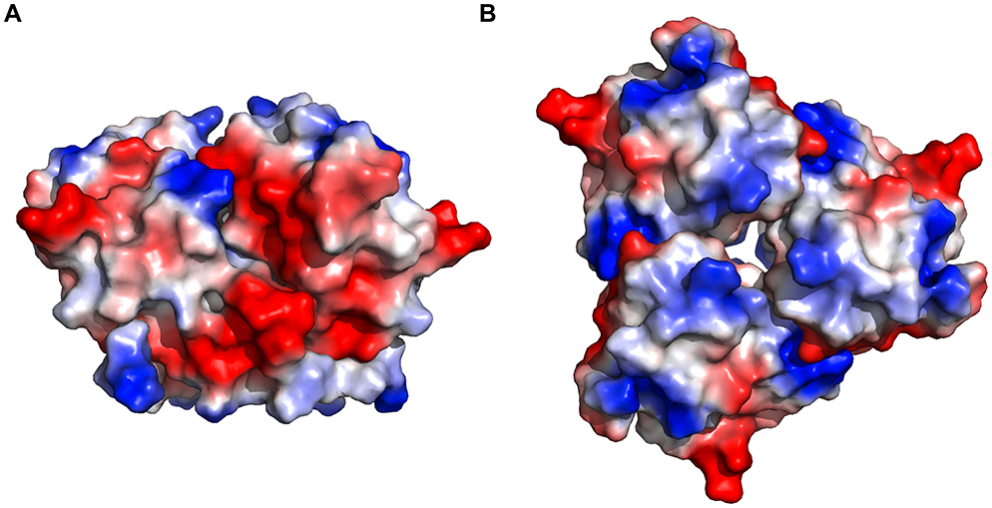
Qualitative electrostatic surface diagram of the SnAdV-1 fibre head. A. Side view in the same orientation as Fig. 1C. B. Top view in the same orientation as Fig. 1D.

Although surface-exposed loops play the most important roles in adenovirus fibre-receptor interaction, surface charge may also have an effect [9], [53]. Positively charged patches are naturally inclined to bind negatively charged molecules such as sialic acid, which is a common terminal glycan on cell surface proteins. The surface of the SnAdV-1 fibre head is mixed in its predicted charge distribution and extensive positive patches are absent (Fig. 3). There is an extended negatively charged patch on the side of the trimer, however, no known positively-charged adenovirus receptors have been described. Most of the specific residues that bind the known human adenovirus receptors CD46 [56] or desmoglein-2 [57] are absent in the SnAdV-1 fibre head. Based on the HAdV-11 co-crystal structure with CD46 [56], Arg279, Arg280, Asp284 and Gln305 are the most important residues for interaction. Of these, only Asp284 may be conserved in the SnAdV-1 fibre head (Asp319). Furthermore, the DG- and HI-loops are in a very different orientation. Amino acids which have been shown to be important for desmoglein-2 binding with HAdV-3 and HAdV-14 [57] are also not conserved in the SnAdV-1 fibre head. Finally, the SnAdV-1 fibre head domain does not contain putative heparan sulphate binding sequences [58], although a KKIK sequence at the very amino-terminus (residues 2–5) of the fibre could play this role.

Extensive surface loops present on fibre heads of other adenoviruses may favour the possibility of altering the antigenic nature of the fibre head by mutation; the relative absence of loops suggests that SnAdV-1 and perhaps Atadenoviruses in general have less need for this mechanism. Knowledge of the snake immune system is limited, although, like in mammals, it consists of innate, cell-mediated and humoral mechanisms [59]. Since reptiles are ectotherm organisms, their immune system is highly sensitive to temperature or seasonal changes. Perhaps viruses like SnAdV-1 can profit from less favourable conditions to avoid interactions with components of the immune system.

In conclusion, the fibre head domain of Snake Adenovirus 1 is the first Atadenovirus protein for which the atomic structure has been solved. The compact trimeric structure is very stable as a result of extensive non-covalent interactions between its monomeric units, forming the trimer. The structure is the smallest among known adenovirus fibre head structures, while it retains the same overall beta-sandwich topology and may therefore be seen as a “minimal” adenovirus fibre head fold. The structure is even more similar to the head domains of certain dsDNA bacteriophage receptor binding proteins and the dsRNA-virus (reovirus) fibres, which prompts the question whether this similarity is the result of divergent or convergent evolution, or perhaps horizontal gene transfer events.

An important application of adenoviruses is their use as vectors for human gene and cancer therapy and for vaccination purposes. Animal adenoviruses may be of special interest due to the lack of neutralizing antibodies in human sera against them. The detailed structure of the SnAdV-1 fibre head and other animal adenovirus fibre heads, together with future identification of their natural receptors, may lead to the development of new strategies to target adenovirus vectors to cells of interest.

## Supporting Information

S1 Figure **Packing of the** ***F*** **23 space group crystal.** A. Bottom view. One SnAdV-1 fibre head trimer is shown in cyan, orange and blue. Asterisks indicate the amino-terminal Glu232, while a hash sign indicates where the shaft domain is expected to be. B. Side view of A. There is not enough room for a shaft domain in the crystal packing; furthermore, the amino-terminal ends of the structure point away from each other and from the three-fold symmetry axis where the shaft domain is expected to be.
(TIFF)

S2 Figure **Disorder prediction (DISEMBL program).** A peak in predicted disorder probability between residues 230 and 236 suggests there may be some flexibility between the putative shaft domain and the head domain.
(TIFF)
