# Correction: Crystal Structure of the Fibre Head Domain of the Atadenovirus Snake Adenovirus 1

**DOI:** 10.1371/journal.pone.0120613

**Published:** 2015-03-25

**Authors:** The PLOS ONE Staff

There is an error in the Data Availability section and the Materials and Methods section. The correct accession code for I213 selenomethionine derivative is 4D0U.

The complete, correct Data Availability section is as follows:

The authors confirm that all data underlying the findings are fully available without restriction. Coordinate files and structure factors are available from the PDB under codes 4D0U (I213 selenomethionine derivative), 4D1F (first P212121 crystal form), 4D1G (second P212121 crystal form), 4D0V (I213 native) and 4UMI (F23 crystal form).

The sixth sentence in the second paragraph of the Materials and Methods section should read as follows: Coordinate files and structure factors will be available from the PDB under codes 4D0U (I213 selenomethionine derivative), 4D1F (first P212121 crystal form), 4D1G (second P212121 crystal form), 4D0V (I213 native) and 4UMI (F23 crystal form).
