# Supplementary figures and images for: Crystal Structure of the Fibre Head Domain of the Atadenovirus Snake Adenovirus 1
Source: PLoS One. 2014 Dec 8;9(12):e114373. doi: 10.1371/journal.pone.0114373 (PMC4259310; doi:10.1371/journal.pone.0114373)

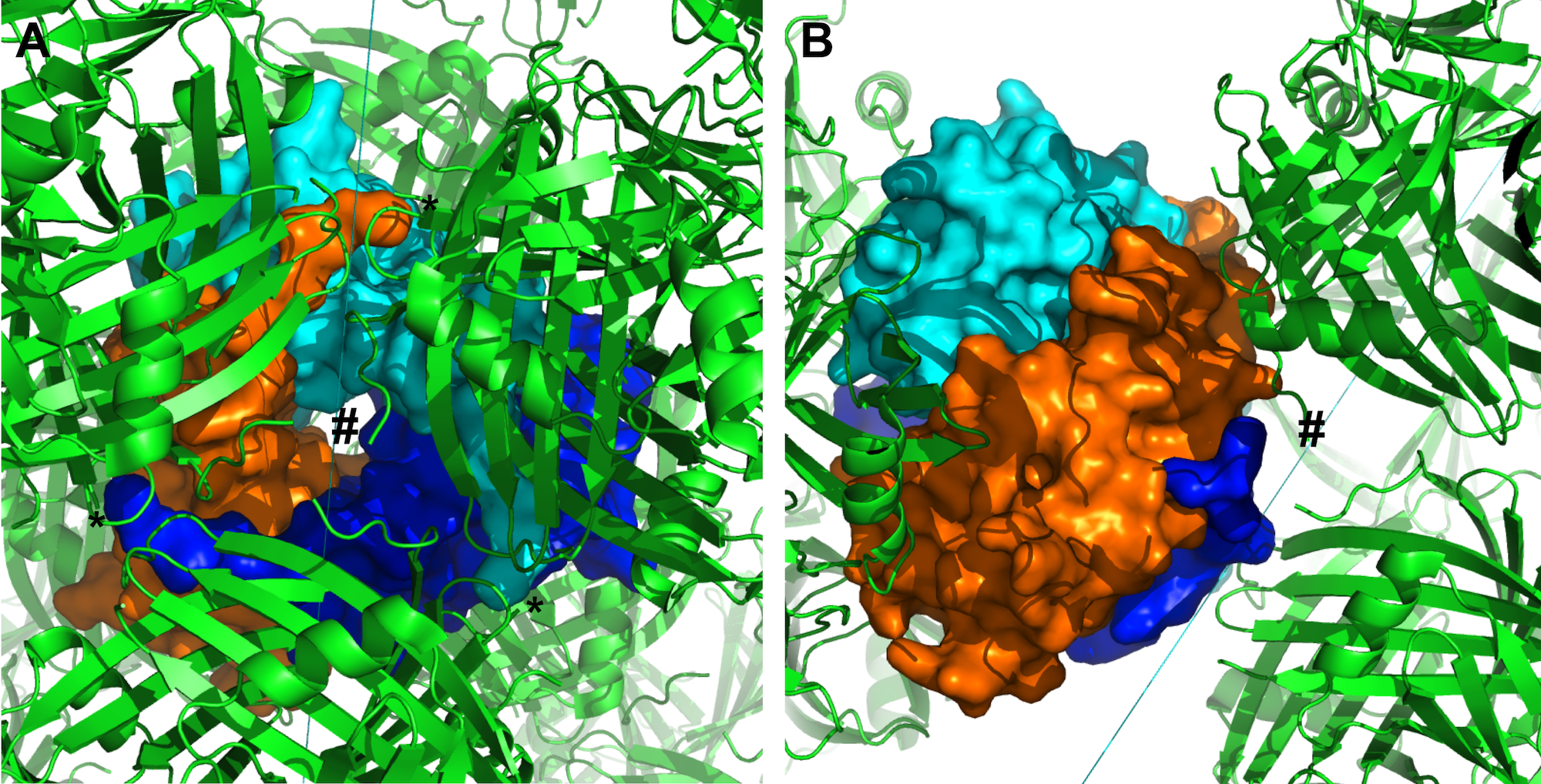

Supplement: S1 Figure — Packing of the F 23 space group crystal. A. Bottom view. One SnAdV-1 fibre head trimer is shown in cyan, orange and blue. Asterisks indicate the amino-terminal Glu232, while a hash sign indicates where the shaft domain is expected to be. B. Side view of A. There is not enough room for a shaft domain in the crystal packing; furthermore, the amino-terminal ends of the structure point away from each other and from the three-fold symmetry axis where the shaft domain is expected to be. (TIFF) [file pone.0114373.s001.tiff]

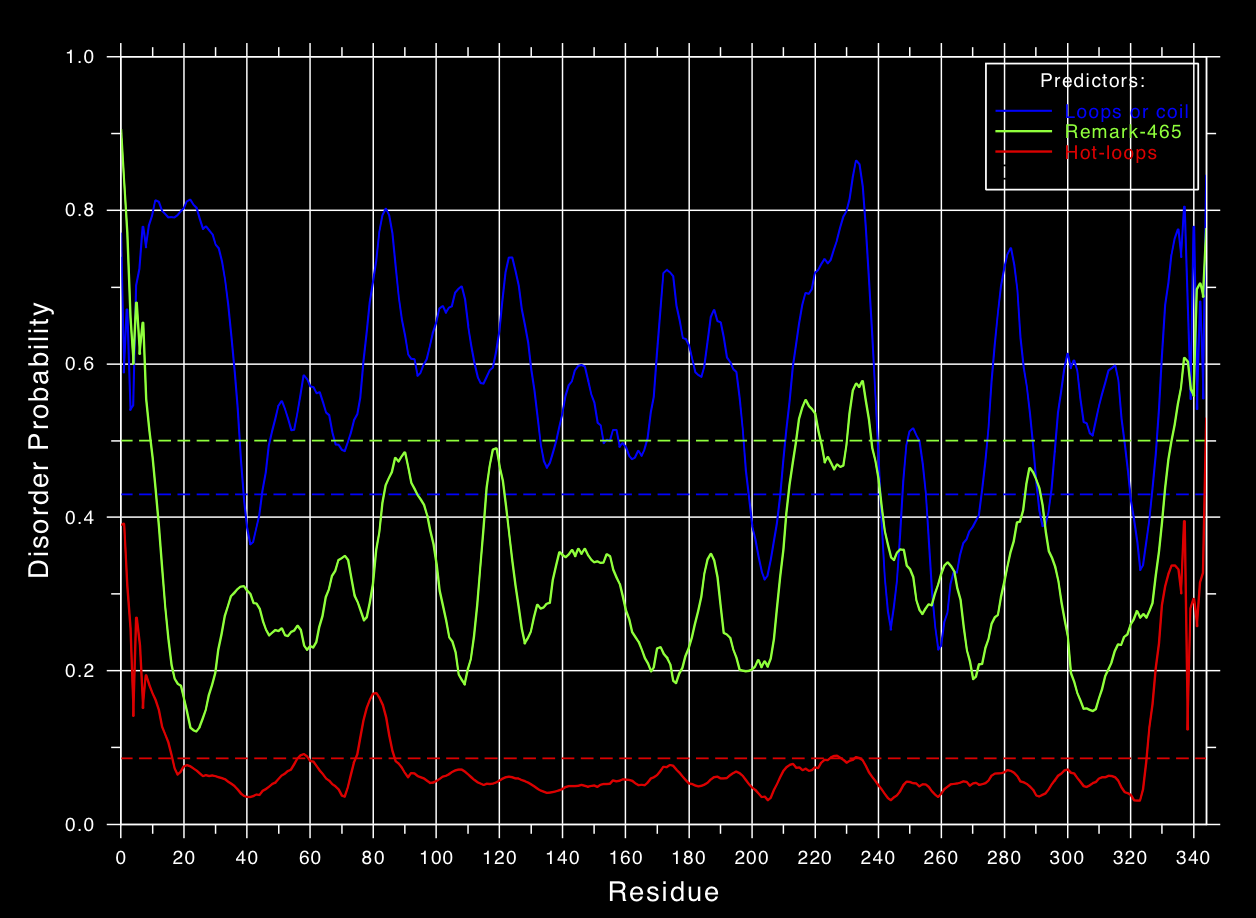

Supplement: S2 Figure — Disorder prediction (DISEMBL program). A peak in predicted disorder probability between residues 230 and 236 suggests there may be some flexibility between the putative shaft domain and the head domain. (TIFF) [file pone.0114373.s002.tiff]
